# The effect of asset-based wealth inequality on problem drinking among rural Thai elders: A prospective population-based cohort study^☆^

**DOI:** 10.1016/j.socscimed.2013.09.025

**Published:** 2014-01

**Authors:** Tawanchai Jirapramukpitak, Melanie Abas, Kanchana Tangchonlatip, Sureeporn Punpuing

**Affiliations:** a Department of Psychiatry, Thammasat University, Faculty of Medicine, Paholyothin Road, Klong Luang, Pathumthani 12120, Thailand; b Section of Epidemiology, Institute of Psychiatry, King's College London, DeCrespigny Park, London SE5 8AF, UK; c Institute for Population and Social Research, Mahidol University, Salaya, Phutthamonthon, Nakhon Pathom 73170, Thailand

**Keywords:** Inequality, Alcohol-related disorders, Aged, Thailand, Longitudinal

## Abstract

Evidence on the link between income inequality and alcohol-related problems is scarce, inconclusive and dominated by studies from the developed world. The use of income as a proxy measure for wealth is also questionable, particularly in developing countries. The goal of the present study is to explore the contextual influence of asset-based wealth inequality on problem drinking among Thai older adults. A population-based cohort study with a one-year follow-up was nested in a Demographic Surveillance System (DSS) of 100 villages in western Thailand. Data were drawn from a random sample of 1104 older residents, aged 60 or over (one per household) drawn from all 100 villages, of whom 982 (89%) provided problem drinking data at follow-up. The primary outcome measure was a validated Thai version of the Alcohol-Used Disorder Identification Test for problem drinking. Living in areas of high wealth inequality was prospectively associated with a greater risk for problem drinking among older people (adjusted odds ratio 2.30, 95% confidence intervals 1.02–5.22), after adjusting for individual-level and village-level factors. A rise in wealth inequality over the year was also independently associated with an increased risk of problem drinking (adjusted odds ratio 2.89, 95% confidence intervals 1.24–6.65). The associations were not explained by the social capital, status anxiety or psychosocial stress variables. The data suggest that wealth inequality and an increase in inequality across time lead to a greater risk of problem drinking. Efforts should be directed towards reducing gaps and preventing large jumps in inequality in the communities. Further research should investigate the effect of asset-based inequality on various health risk behaviors and its specific mediating pathways.

## Highlights

• Evidence on inequality and alcohol-related outcomes is inconclusive.
• Assets may be more reliable for measuring inequality in developing countries.
• The findings suggested that wealth disparity increased the risk of problem drinking.
• Widening disparity across time also increased the risk.
• To address problem drinking may need to target areas with high wealth inequalities.

## Introduction

Alcohol-related problems are a major global public health concern being associated with a wide range of social and economic challenges (WHO, 2007b). Alcohol is a major contributor to global burden of disease and the third most important health risk factor, leading to premature death and disability (WHO, 2009). From 1997 to 2007 the amount of alcohol consumption among Thai people has been steadily increasing from 7.28 L of pure alcohol per head to 7.71 L per head (WHO, 2007a). Alcohol is also the largest health risk factor contributing to 8.1% of the total burden of disease from all causes (in Disability Adjusted Life Years or DALYs) in Thailand (Thai Working Group on Burden of Disease, 2011).

The notion that the distribution of wealth within societies could be a health determinant has attracted a great deal of attention in public health research. Thailand is a developing country which has seen significant growth in Gross Domestic Product (GDP) over the past few decades. However, this economic growth has been unequal across groups of populations with greater increases for the rich, thereby contributing to greater disparity (Phongpaichit, 2011). Thailand's GINI coefficient of household income inequality, an index measuring the extent to which the distribution of income among households within an economy deviates from a perfectly equal distribution, is estimated to be 0.54 in 2009 (CIA, 2009), which is among the highest in the world. By comparison, most developed countries have a GINI under 0.40 (UNDP, 2009). Income inequality has been shown to be associated with poorhealth and social outcomes including all-cause mortality (Ross et al., 2000), violent crime (Hsieh & Pugh, 1993) and substance use (Galea, Ahern, Tracy, & Vlahov, 2007). The impact of income inequality on life expectancy at birth is also observed in Thai people (Rojroongwasinkul, 2006).

A number of theories have been put forward to explain the association between wealth inequality and health. For instance, the ‘social capital’ theory holds that higher levels of wealth inequality increase status differentials between individuals, thereby reducing levels of interpersonal trust (Kawachi & Berkman, 2000; Kennedy, Kawachi, & Prothrow-Stith, 1996). The ‘status anxiety’ theory on the other hand argues that inequality damages individual health via psychosocial processes based on perceptions of one's place in the social or status hierarchy (Wilkinson, 1996, 2005). The perception of inferiority may produce negative feelings which damage individual health via psycho-endocrine mechanisms but also damage health and well-being indirectly by reducing levels of social capital within society (Wilkinson & Pickett, 2009). The ‘status anxiety’ model therefore shares the psychological stress mechanism with the ‘social capital’ hypothesis. Inequality may put people under stress through such mechanisms and make them more likely to adopt stress-relieving behaviors such as drinking and substance use (Rhodes & Jason, 1990).

Wealth inequality can be measured in a number of ways, but is most often measured through the GINI coefficient of income inequality in the field of health research (Kondo et al., 2009). The use of income as a proxy measure for wealth in developing countries remains problematic because data on income are often unavailable or unreliable. Moreover, in some circumstances, inequality of assets may be more prominent than income inequality and more relevant to economic development (Deininger & Olinto, 2000; McKenzie, 2005). For example, it is well-known that income in kind (e.g., from home production) is of particular importance in developing countries. If people rely disproportionately on income in kind, its neglect might lead to significantly inaccurate inequality. In addition, inequality measures should be representative of the population at large. Restricting attention to income may be acceptable in developed economies where wage earners comprise the lion's share of the economically active population. In developing countries, however, a significant share of the population is self-employed in agriculture or in informal sector, and has various sources of income (e.g., remittances), which are often uncertain and cannot be easily corrected for. Because information on certain kinds of asset is usually easy to obtain and appears to be a more reliable and valid measure of wealth than the measure of income or expenditure, the asset index has been introduced to address such problems (Falkingham & Namazie, 2002; Prakongsai, 2006) and later led to the development of an asset-based approach to measuring wealth inequality (McKenzie, 2005).

In measuring inequality, it is recommended that choosing a unit of analysis to capture the right level of differentiation should be on the basis of the processes through which inequality may influence specific health outcomes (Diez Roux, 2001). In previous studies on income inequality and health, the choice is often a large-scale geographical unit such as regions or countries. However, measuring inequality at a local level may also be relevant, particularly in developing countries, where a large section of the population live in small villages in rural areas. The perception of inequality may be salient at the village level because people living in the same village are more likely to be closely connected and influenced by their neighbors. Comparisons of wealth with neighbors, the resulting sense of inferiority or disparities in achievement, may all collude to create anxiety in a small village environment. However, the effect of inequality can also operate at the neighborhood level through social-interactional and institutional mechanisms, which in turn account for a variety of problem behaviors (Sampson, Morenoff, & Gannon-Rowley, 2002). In Thailand, a village is also an administratively defined neighborhood and relevant when local health and social policies are involved.

To date, studies examining the link between wealth inequality and alcohol-related problems, all carried out in the United States and Europe, have shown inconsistent findings (Blomgren, Martikainen, Makela, & Valkonen, 2004; Elgar, Roberts, Parry-Langdon, & Boyce, 2005; Galea et al., 2007; Henderson, Liu, Diez Roux, Link, & Hasin, 2004). A study in New York found that maldistributed neighborhood income was significantly associated with a greater likelihood of alcohol use (Galea et al., 2007). Another survey conducted in countries in Europe and America found that adolescents of certain age groups in countries of high income inequality consumed more alcohol and reported more episodes of drunkenness than their counterparts in countries of low income inequality (Elgar et al., 2005). Other studies, however, did not find the effect of inequality at a state- or regional-level on alcohol outcomes among general adult populations (Blomgren et al., 2004; Henderson et al., 2004). Such a contextual influence of wealth inequality has not yet been explored in low or middle-income developing countries, which are generally more likely to suffer from more disparity.

Kanchanaburi's Demographic Surveillance System (DSS) presents a unique opportunity to study the prospective relationship of wealth inequality and health outcomes. Kanchanaburi is a large province in western Thailand and the only one in the country which has a DSS, comprising a longitudinal census for monitoring population changes. DSS is especially suited for studies on older people because they rarely migrate and are less likely to be lost during follow-up compared to younger people. In 2007, about 11% of those aged 60 years or over in Kanchanaburi had reported drinking alcohol (Center for Alcohol Studies, 2007).

Our goal in the present study was to examine the prospective relationship between asset-based wealth inequality at baseline, its one-year change and risk for individual problem drinking among older people aged 60 or over at one year follow-up, accounting for individual- and village-level potential confounders. We also sought to examine the possible mediating effect of individual-level perceived social capital, social anxiety and psychological stress variables.

## Methods

### Study design and setting

We obtained information from a data source originally designed to investigate out-migration of children and its impact on mental health of the older parents left behind in rural Thailand (Abas et al., 2009), nested in the Kanchanaburi Demographic Surveillance System (DSS) in western Thailand (Institute for Population and Social Research, 2001). The DSS provides a longitudinal database on over 12,500 households (containing over 42,000 individuals) in 100 villages (or sampling units), which were selected from a total of nearly 1000 villages. The sampled villages comprised 20 from each of 5 strata; rice producing (20/193), plantation crops (20/93), upland areas (20/94), mixed economy (20/491) and urban/semi-urban (20/133). These communities have consented to taking part in annual data collection since 2000.

The sampling strategy had been developed for the main study (Abas et al., 2009), based on whether or not older adults had children living with them. The sample size was based on a comparison of prevalence of depression in those without any children living nearby versus those with some children living nearby and required a total sample size of 954, given the proportions of depression expected of those exposed and not exposed to having all their children living away from the district. Because older adults who lived without any children nearby were relatively uncommon, we chose to oversample this particular group. We therefore sampled 30% of households in each sampling unit where an adult aged ≥ 60 years was living with at least one of their children in the same household and 60% of households where an adult aged ≥ 60 years was not living with at least one of their children in the same household. We excluded those older adults without at least one living child – biological, step-child or adopted (fewer than 5% of those over 60 in this setting), and those for whom Thai was not the household language (less than 1%). There were no other exclusions. We used random selection to select the participant in situations where there was more than one eligible older person living in a household. Baseline assessment interviews were carried out from November to December 2006 in the respondents' homes. We aimed to re-interview all respondents 1 year after their baseline assessment. The follow-up interviews were completed between November and December 2007. From the potential eligible sample of 1300 older adults in 1300 households, 1104 (84.9%) provided data at baseline (110 were unavailable for interview despite up to 10 visits to the households, 21 refused and 65 were either unwell and/or cognitively impaired at interview). There were no significant differences with non-responders in terms of age, gender, living alone, marital status or education.

### Individual-level variables

The tension reduction model, a widely known model of drinking and alcoholism (Conger, 1956), was used and adapted as the basis for selecting variables potentially acting as confounders. Basically, the model hypothesizes that people drink alcohol as a means to relieve stress or tension. Stressors are therefore considered to be a major risk factor for problem drinking. A variety of acute and chronic adversities such as divorce, poor education, unemployment, low socioeconomic status and other life stressors have previously been reported to be risk factors for problem drinking (Assanangkornchai, Sam-Angsri, Rerngpongpan, & Lertnakorn, 2010; Frijters, Johnston, Lordan, & Shields, 2013; Galea et al., 2007; Kinra et al., 2010; Peltzer & Phaswana-Mafuya, 2013; Veenstra et al., 2007). Higher rates of such adversities are also reportedly observed in areas of high levels of inequality (Björklund, 1991; Levine, Frank, & Dijk, 2010; Pickett & Wilkinson, 2007; Wilkinson & Pickett, 2009). In light of these findings, the present study included age, sex, marital status, educational attainment, working status, stressful life events, household wealth index and problem drinking, which were assessed twice at the times of the interview.

Educational attainment was categorized in years; 0 (no education), 1–3 (not completing compulsory education), 4 (completing compulsory education), 5–13 (higher than compulsory education).

Household wealth index: The heads of household were asked to report the presence of household assets on the list provided (up to 24). We used principal component analysis to develop a household wealth index comprising 15 household assets (namely, a television set, video player, stereo, telephone, computer, water pump, air conditioning, sewing machine, washing machine, bicycle, fridge, motorcycle, mobile phone, car and pick-up truck) and interviewer's global rating of household quality. Scoring factors of the first principal component was used to construct the asset index of each household. The mean value of the index is zero by construction. Household asset index has been validated and widely used in many developing countries as proxy for wealth (Falkingham & Namazie, 2002; Prakongsai, 2006).

*Problem drinking* was assessed using the Alcohol Use Disorder Identification Test (AUDIT), a structured and standardized instrument which provides valid and reliable detection of hazardous and harmful use of alcohol in a general population (Saunders, Aasland, Babor, de la Fuente, & Grant, 1993). It has been validated and used in clinical studies and community surveys in Thailand (Assanangkornchai et al., 2010; Jirapramukpitak, Prince, & Harpham, 2008; Lapham et al., 1998). A cut-off score of 8 or above indicates problem drinking.

*Perceived social position* was assessed by items taken from the Thai well-being scale (Ingersoll-Dayton, Saengtienchai, Kespichayawattana, & Aungsuroch, 2001, 2004), which can be used to examine the status anxiety pathway. The scale has five dimensions, one of which has three items measuring ‘respect from others’. The participant was asked to indicate on a 4-point scale if each of the statements below was not at all true, slightly true, somewhat true or very true:

• Younger members of your extended family or other young people obey you.
• Younger members of your extended family or other young people talk and behave politely toward you.
• Younger members of your extended family or other young people treat you with respect.

For analysis, the scores were summed and divided into high/low according to the median.

*Social trust and support* was measured by six items taken from the Thai well-being scale. They were used to assess trust among neighbors and social support from neighbors and extended family, a proxy for the social capital pathway. The participant was also asked to indicate on a 4-point scale if each of the statements below was not at all true, slightly true, somewhat true or very true:

• In your extended family, people get along well together
• Members of your extended family care about each other.
• In your extended family, people depend on each other for help.
• People in your extended family take care of you.
• In your neighborhood, people are friendly to each other.
• Neighbors depend on each other.

The scores were then summed and divided into high/low using the median cut-off.

*The Hospital Anxiety and Depression Scale (HADS)* is a 14-item instrument designed to measure anxiety and depression symptomatology (Zigmond & Snaith, 1983). The HADS has been extensively validated in a variety of adult populations, including clinical and community samples. The good reliability and stability of the HADS were also demonstrated in various translated versions across culturally diverse groups (Caci et al., 2003; Herrmann, 1997; Mykletun, Stordal, & Dahl, 2001; Nilchaikovit, Lortrakul, & Paisansuthideth, 1996). Participants were asked to complete the scale by rating how they felt on the basis of symptoms that had occurred in the preceding week using a 4-point scale, ranging from 0 to 3 (0: absence of symptoms, 3: severe symptoms). In the present study, the 7-item anxiety sub-scale was used as a proxy for the psychological stress pathway. Those who scored more than 11 on the subscale were regarded as having significant psychological stress.

### Neighborhood definition

Kanchanaburi is divided into 13 districts, which are divided further into 95 subdistricts called “Tambon” and further into 959 villages. In the Kanchanaburi DSS, a village on average consists of 125 households (range 25–337). A household has an average of 4 residents (Yoddumneun-Attig, 2004). These villages were sampled and used as neighborhood units in the analyses.

### Village-level predictor variables

Our main predictor variables were village wealth, wealth inequality and their one-year changes. These were measured for each village where a respondent resided at the times of the interview from baseline to follow-up. Village wealth was simply the median of the household asset scores among the sampled households in each village. It was classified for analysis into high and low levels of wealth based on the median cut-off. We used the relative measure of inequality as the measure of wealth inequality on the basis that data from on household infrastructure, building materials, and ownership of certain durable assets can be used to measure inequality in living standards (McKenzie, 2005). The measure was defined as the standard deviation of the first principal component in a given community of interest relative to the standard deviation in the sample as a whole. Where *y* is the first principal component of the listed assets, relative measure of inequality for community *c* is calculated by the following formula.

$$I_{c}=\frac{\sigma_{c}}{\sqrt{\lambda }}$$

where *σ*_*c*_ is the sample standard deviation of the *y*_*i*_ across households in community *c*, and *λ* is both the eigenvalue corresponding to the first principal component, and also the variance of *y*_*i*_ over the whole sample. *I*_*c*_ will be greater than one if community *c* displays more inequality within it than does the sample population as a whole. In the present study, the sample villages were simply equally divided into 2 groups according to their level of wealth inequality: villages of high and low inequality.

### Data collection procedure

There were eight interviewers, all trained and supervised by the research team in Thailand. All the interviews were conducted in Thai and informed consent was obtained from all participants. If the selected older adults and the head of household gave consent, the interviewer first interviewed the head of household with the household questionnaire and then the older adult with the individual questionnaire. Quality control included checking on data completeness and consistency. Interviewers had to return to the participants if the data were incomplete. We gained ethical approval from King's College Ethics Committee (No. 05/05-68) and from Mahidol University Institutional Review Board.

### Statistical methods

We describe the sample at baseline and at follow up using means and standard deviations or percentages as appropriate. We first examined the prevalence of problem drinking at follow-up for each category of risk factors and used the chi-square test to detect bivariate associations between problem drinking and risk factors. Student's *t* test and Mann–Whitney *U* test were used to evaluate statistically significant differences in continuous variables where appropriate. We then used multilevel logistic regression models, to analyze the prospective relationship between wealth inequality, as well as its one-year change, in village of residence and individual AUDIT outcome as the dependent variable. Because individuals were clustered within villages and villages within strata, three-level models with variances at the level of individual, village and strata were used. The model was a random intercepts model in which the regression coefficients of the independent variables were assumed to be the same for all areas (fixed effects), but the intercepts were allowed to vary across areas (random effects) i.e., village and strata in the present study. The analysis was not extended to random slope models. All risk estimates, represented by odds ratios (OR), and their 95% confidence intervals (CI) were calculated using STATA version 10 for Windows (StataCorp, 2007).

## Results

### Response rate and loss to follow-up

Out of the 1300 eligible older people at baseline, 153 (11.8%) were non-responders of whom 110 were unavailable for an interview (despite up to ten visits to the household), 21 refused to take part and 22 were too unwell. Those unavailable were mostly away visiting their children. Of the 1147 (88.2%) who agreed to participate, data were complete for 1104 and incomplete for 43 because the older adults were unwell or cognitively impaired. Of 1104 respondents who formed the baseline sample, 982 (89%) provided complete data on AUDIT and on other key variables at one-year follow-up. The main reasons for not completing any follow-up measures were absence 39 (32%) despite up to 3 visits, illness 14 (11.5%) and death 34 (27.9%), 28 (23%) had moved and could not be traced and only 7 (5.7%) refused to take part. Although several characteristics were individually associated with missing follow-up, older age, cognitive impairment and disability remained significant predictors in multiple regression. In the end, a total of 96 villages were used for analyses. People in the remaining 4 villages were too few (*n* = 1) or unavailable at follow-up (*n* = 3). The median number of participants in each village was 9 (range 3–31).

### Characteristics of sample

The mean age of the study participants at baseline was 69.4 years (sd 6.86). The majority of the sample was female, married, currently in work, and completed four years of education (Table 1). Characteristics of the sample were comparable to characteristics of the general elderly population obtained from the Kanchanaburi DSS in 2004, in terms of age and gender compositions (Yoddumneun-Attig, 2004). About 42% of the sample reported having one or more stressful life event. Among the most common life events reported were having serious financial problems (27.5%), serious problems with getting health care for their close ones (14.2%), and serious problems with illness, injuries or disability of theirs, their partners or children (8.5%). Just over half of the sample experienced a decrease in household wealth across the year. The median village wealth score was −0.10 (range −3.20 to 3.32) and around one-third of the sample experienced a decrease in village wealth during the one-year period. The mean of the relative inequality measure was 0.42 (sd 0.09, range 0.18–0.71). The average one-year change in inequality across the villages was −0.02 (sd 0.09, range −0.54 to 0.21), amounting to an overall reduction of 4.38%. Around 41% of the sample experienced an increase in wealth inequality over the one-year period. The prevalences of problem drinking were 6.9% and 5.3% at baseline and at follow-up, respectively.

### Characteristics of sample at baseline and association with problem drinking at follow-up: bivariate analysis

Problem drinking at follow up was significantly associated with many of the baseline characteristics, including younger age, male gender, married or single/divorced/separated status and being currently in work (Table 1). Those with problem drinking at follow-up had a significantly lower asset score than those without problem drinking (*t*-statistic = 2.151, *p* = 0.0159). Those with and without problem drinking had no significant differences in level of village wealth (*U*-statistic, *p* = 0.176) and level of inequality at baseline (*t*-statistic = −0.462, *p* = 0.322). All the mediating variables including social capital, status anxiety and psychological stress were not significantly associated with problem drinking.

### Village wealth, wealth inequality and problem drinking at follow-up: multivariate analysis

The multilevel logistic regression analyses showed that at the individual level, problem drinking at baseline, younger age and male gender remained significantly associated with risk for problem drinking at follow-up. Village-level wealth at baseline was not significantly associated with increased risk for problem drinking at follow-up, both before and after adjustment for the individual-level and village-level confounders (Table 2). However, living in areas of high inequality at baseline was significantly associated with greater risk of problem drinking at follow-up, after adjusting for all the potential confounders (OR 2.30 95% CI 1.01–5.20). In addition, living in areas with an increase in wealth inequality was also prospectively and independently associated with risk for problem drinking (OR 2.87 95% CI 1.24–6.64) (Table 3). There was no statistically significant variance in the intercepts of different villages and strata. To assess whether there were graded relationships between amount of inequality and problem drinking, a multivariate analysis with the level of inequality at baseline and its one-year changes (in tertiles) was presented (Table 4). No significant linear trend in risk for problem drinking was observed across the groups of low, medium and high inequality at baseline (*p* = 0.141). However, there was a significant trend towards greater risk of problem drinking among those experiencing varying changes, from a decrease to a rise, in inequality (*p* = 0.009). Table 5 showed that the observed associations between wealth inequality and problem drinking were not mediated by any of the social capital, status anxiety and psychological stress variables.

## Discussion

Our findings demonstrate a contextual effect of village wealth inequality on risk for individual problem drinking among older people at one-year follow-up after controlling for a set of potential individual- and village-level confounders. In addition, a strong prospective relationship between one-year growth in inequality and problem drinking was observed. This finding was further strengthened with the presence of a gradient in risk for problem drinking among those exposed to varying changes, from a decrease to a rise, in inequality. In other words, older adults living in areas of high and/or growing wealth inequality had a significantly greater risk for problem drinking. Wealth at the household and village levels, on the other hand, was not found to be associated with problem drinking. Previous studies examining the contextual effect of income inequality on individual drinking outcomes have reached different conclusions, although they are not exactly comparable due to differences in age groups under study, alcohol outcomes and unit of analysis (Blomgren et al., 2004; Elgar et al., 2005; Galea et al., 2007; Henderson et al., 2004). Our study findings seem rather in line with two studies, which found contextual effect of income inequality on particular alcohol outcomes among a general adult population or at least among certain age groups (Elgar et al., 2005; Galea et al., 2007). Important to note is that the two studies had a rather wide variation in GINI values across their geographical units of analysis and also included areas with GINI values in a high range for comparison. In contrast, studies which found no effect of income inequality on alcohol outcomes had a relatively limited range of GINI across units of analysis and/or only included GINI values in a low range (Blomgren et al., 2004; Henderson et al., 2004). Thus, one possible explanation for the mixed pattern of findings observed for wealth inequality and alcohol outcomes is that a small variation in inequality under study or in intra-country income inequality in some countries (such as Scandinavian nations) may lead to unnoticeable effects (Lynch et al., 2004). Another possibility is a “threshold” effect of wealth inequality on alcohol outcomes. Evidence suggests that areas with GINI values greater than 0.3 show a more consistent association between income inequality and adverse health outcomes (Kondo et al., 2009). Incidentally, the fact that in the present study we did not find a significant linear trend in risk for problem drinking across groups with low, medium and high levels of inequality at baseline and that the size of the association with baseline inequality began to grow substantially, though non-significant, in the highest inequality group, may suggest a threshold effect of inequality.

Our findings showed that the hypothesized perceived social capital, status anxiety and psychological stress pathways were not supported by our data. Along a similar line, a recent study investigating the neighborhood effect on hazardous alcohol use found that the relationship between high neighborhood disorder and more hazardous alcohol use was not mediated by individual psychological distress (Kuipers, van Poppel, van den Brink, Wingen, & Kunst, 2012). The question remains whether the mechanisms linking inequality and alcohol outcomes operate at either individual or neighborhood level or both. Because the present study cannot exclude the possibility of neighborhood-level pathways, future studies should investigate specific mechanisms operating at such level, such as social-interactional and institutional mechanisms (Sampson et al., 2002). On the other hand, the neomaterialist hypothesis, which is also regarded as a contextual explanation, may also provide another potential avenue for further investigation. Neomaterialists argue that societies that allow wealth inequality to get bigger may also tend to be the ones that underinvest in public goods such as social infrastructure and services that promote physical and mental health (Kawachi, Kennedy, Lochner, & Prothrow-Stith, 1997; Wilkinson, 1996). This may in turn contribute to a lack of social resources available to overcome problem drinking once initiated (Galea et al., 2007).

## Limitations and strengths

The present study was the first, to our knowledge, to provide evidence of a prospective relationship between asset-based wealth inequality and individual problem drinking. The particular strength of our study lay in the utilization of the fact that high wealth inequality and the continuing rise in wealth inequality were particularly prominent in many developing nations. Another advantage was that the older populations in the present study were largely local and rarely migrated, as opposed to younger adults, hence loss to follow up due to migration was negligible. The contextual effects observed were also therefore likely to be due to having been exposed to social environment in their neighborhood and less likely to be due to the effect of selective migration, a kind of bias where migrants with certain characteristics choose to live in a particular place and influence their environment, rather than vice versa. The results of the study, however, should be interpreted with caution. The overall sample was relatively small and the prevalence of problem drinking rather low. All these may contribute to the marginally significant contextual effect of high inequality at baseline on problem drinking, which came out as such only after controlling for the full set of potential confounders. Thus, the role of chance as a possible explanation for the observed finding could not be totally excluded. However, this was unlikely because the size of the association changed very little after adjusting for a series of potential confounders, from the basic model controlling for age, gender and baseline problem drinking only (OR 1.9) (data is not shown), to Model I (OR 2.00) and to Model II (OR 2.03). Only when controlling for changes in wealth from baseline to follow up did the association slightly strengthen (OR 2.3) and became significant. In other words, controlling for more variables did little in strengthening the association. The likelihood of chance was reduced further still by the fact that strong contextual effect with changes in inequality was also observed in the present study. Nevertheless, future studies should benefit from additional data collected from a larger sample size, which may include younger age groups, because they are more likely to have a high prevalence of problem drinking by comparison. A more accurate picture of such contextual effect can also be obtained from young problem drinkers, who have only recently developed such behavior. It is possible that that current problem drinking among older people, like many mental illnesses or chronic diseases, runs a remitting and relapsing course, and may also be determined more by earlier characteristics of the life course, and less by recent exposure to a certain environment.

Consideration should be taken into account when considering whether the current findings can be generalized to other settings. On the other hand, including only older adults in the sample should not limit the validity of the study findings. It could be argued that findings that are replicated in any specific age group can be seen as further evidence that supports the general theory on inequality and health.

It should be noted that the manner in which the mediators are operationalized in this article are far from ideal. There may be better candidates for the variables we used to represent the hypothesized pathways in the present study, particularly the relatively complicated social capital concept. To address this issue, a priori and validated measures of such pathway mediators should be used.

## Implications

Measuring inequality based on a wide variety of assets may be more appropriate than income inequality in developing countries. To reduce problem drinking, policy makers may need to be concerned more about households' access to assets and the associated opportunities. Long-term strategies to curb problem drinking should also target those in areas where inequalities are high and focus on efforts to reduce gaps and prevent large jumps in asset inequality in the communities. The findings also suggest that interventions to address psychological stress may help little to reduce problem drinking among older people.

## Figures and Tables

**Table 1:** The associations between characteristics of the sample and problem drinking.

Characteristic at baseline	Total *N* (%)	Problem drinking at follow-up		
		Absent *N* (%)	Present *N* (%)	*p*-Value
**Age**				<0.001
60–69	547 (55.7%)	505 (54.3%)	42 (80.8%)	
≥70	435 (44.3%)	425 (45.7%)	10 (19.2%)	
**Sex**				<0.001
Male	436 (44.4%)	395 (42.5%)	41 (78.9%)	
Female	546 (55.6%)	535 (57.5%)	11 (21.2%)	
**Marital status**				0.016
Single/divorced/separated	52 (5.3%)	48 (5.2%)	4 (7.7%)	
Married	557 (56.7%)	519 (55.8%)	38 (73.1%)	
Widowed	373 (38.0%)	363 (39.0%)	10 (19.2%)	
**Work status**				<0.001
Working	504 (51.3%)	465 (50%)	39 (75%)	
Not working	478 (48.7%)	465 (50%)	13 (25%)	
**Years of education**				0.312
0	275 (28.0%)	266 (28.6%)	9 (17.3%)	
1–3	158 (16.1%)	150 (16.1%)	8 (15.4%)	
4	465 (47.4%)	435 (46.8%)	30 (57.7%)	
5–16	84 (8.6%)	79 (8.5%)	5 (9.6%)	
**Stressful life events**				
0	566 (57.6%)	539 (58.0%)	27 (51.9%)	0.604
1	236 (24.0%)	223 (24.0%)	13 (25.0%)	
≥2	180 (18.3%)	168 (18.1%)	12 (23.1%)	
**Wealth index**				
High	493 (50.3%)	473 (50.9%)	20 (38.5%)	0.080
Low	488 (49.8%)	456 (49.1%)	32 (61.5%)	
**One-year wealth change**				
Decrease	520 (53.0%)	497 (53.4%)	23 (44.2%)	0.195
Increase	462 (47.1%)	433 (46.6%)	29 (55.8%)	
**Village median wealth**				
High	505 (51.4%)	480 (51.6%)	25 (48.1%)	0.620
Low	477 (48.6%)	450 (48.4%)	27 (51.9%)	
**One year change in village wealth**				
Decrease	336 (34.2%)	318 (34.2%)	18 (34.6%)	0.950
Increase	646 (65.8%)	612 (65.8%)	34 (65.4%)	
**Wealth inequality**				
Low	487 (49.6%)	465 (50.1%)	22 (42.3%)	0.277
High	494 (50.4%)	464 (50.0%)	30 (57.7%)	
**One-year change in inequality**				
Decrease	581 (59.2%)	555 (59.7%)	26 (50.0%)	0.167
Increase	401 (40.8%)	375 (40.3%)	26 (50.0%)	
**Perceived social position**				
High	450 (46.5%)	424 (46.3%)	26 (50.0%)	0.607
Low	517 (53.5%)	491 (53.7%)	26 (50.0%)	
**Social trust and support**				
High	416 (43.0%)	390 (42.6%)	26 (50.0%)	0.293
Low	552 (57.0%)	526 (57.4%)	26 (50.0%)	
**Psychological stress**				
Absence	930 (94.7%)	883 (95.0%)	47 (90.4%)	0.153
Presence	52 (5.3%)	47 (5.1%)	5 (9.6%)	

**Table 2:** Odds ratios for the associations between wealth variables at baseline and individual problem drinking at follow-up, adjusting for potential confounders.

Variables in the model	Comparison of wealth	*N*	Odds ratio	95% CI		*p*-Value
Model I^a^	High vs. low household wealth	982	0.58	0.27	1.29	0.186
	High vs. low village wealth	982	1.09	0.47	2.56	0.841
	High vs. low inequality	981	2.00	0.85	4.68	0.111
Model II^b^	High vs. low household wealth	981	0.53	0.24	1.20	0.131
	High vs. low village wealth	981	1.24	0.51	3.02	0.638
	High vs. low inequality	981	2.03	0.86	4.79	0.104
Model III^c^	High vs. low household wealth	981	0.65	0.28	1.48	0.301
	High vs. low village wealth	981	1.11	0.47	2.62	0.808
	High vs. low inequality	981	2.30	1.02	5.22	0.046

a Adjusted for all demographic variables: problem drinking at baseline, age, sex, marital status, education, work status, stressful life event and strata.

b Further adjusted for other wealth variables at baseline.

c Further adjusted for changes in household wealth, village wealth and wealth inequality from baseline to follow-up.

**Table 3:** Odds ratios for the associations between changes in household wealth, village wealth and wealth inequality and individual problem drinking at follow-up.

Comparison of wealth change	*N*	Adjusted odds ratio^a^	95% CI		*p*-Value
Increased vs. reduced household wealth from baseline to follow-up	981	1.41	0.68	2.9	0.355
Increased vs. reduced village wealth from baseline to follow-up	981	1.23	0.53	2.82	0.629
Increased vs. reduced inequality from baseline to follow-up	981	2.87	1.24	6.65	0.014

a Adjusted for all the individual- and village-level variables.

**Table 4:** Changes in odds ratios and trends for the associations between inequality (in tertiles) and problem drinking.^a^

	Adjusted odds ratio	95% CI		*p*-value
**Baseline level of inequality**				
Tertile 1: low (from 0.18 to 0.39)	1			
Tertile 2: medium (from 0.39 to 0.44)	1.13	0.41	3.13	0.820
Tertile 3: high (from 0.44 to 0.71)	2.24	0.77	6.59	0.141
**Change in inequality from baseline to follow-up**				
Tertile 1: marked decrease in inequality (from −0.54 to −0.03)	1			
Tertile 2: slight change in inequality in either direction (from −0.03 to 0.01)	2.01	0.69	5.87	0.200
Tertile 3: marked increase in inequality (from 0.01 to 0.21)	4.46	1.48	13.43	0.008

a Adjusted for all the individual- and village-level potential confounders.

**Table 5:** Odds ratios for multi-level logistic model of problem drinking, adjusting for each of the hypothesized pathways.

Comparison of wealth change	*N*	Adjusted odds ratio	95% CI		*p*-Value
**Model 1 (Base)**					
High vs. low inequality	981	2.30	1.02	5.22	0.046
Increased vs. reduced inequality from baseline to follow-up	981	2.87	1.24	6.65	0.014
**Model 2 (Social capital)**					
High vs. low inequality	966	2.30	1.02	5.19	0.046
Increased vs. reduced inequality from baseline to follow-up	966	2.86	1.23	6.67	0.015
**Model 3 (Status anxiety)**					
High vs. low inequality	967	2.28	1.00	5.22	0.050
Increased vs. reduced inequality from baseline to follow-up	967	2.99	1.27	7.05	0.012
**Model 4 (Psychological stress)**					
High vs. low inequality	981	2.30	1.02	5.20	0.046
Increased vs. reduced inequality from baseline to follow-up	981	2.79	1.20	6.49	0.017
